# Two cases of bloodstream infections associated with opportunistic bacterial species (*Enterococcus hirae* and *Enterobacter xiangfangensis*) in companion animals

**DOI:** 10.1186/s12917-023-03615-2

**Published:** 2023-03-25

**Authors:** Raffaele Scarpellini, Massimo Giunti, Alessandra Pontiero, Federica Savini, Erika Esposito, Silvia Piva

**Affiliations:** Department of Veterinary Medical Sciences, University of Bologna, Via Tolara di Sopra 50, Bologna, 40064 Italy

**Keywords:** Bloodstream infections, Bacteremia, Small animal practice, Opportunistic bacteria, *Enterococcus hirae*, *Enterobacter xiangfangensis*

## Abstract

**Background:**

Bloodstream infections are a matter of concern in small animal veterinary practice. Few reports are avaiable, especially regarding the role of opportunistic bacteria in becoming infectious. This report aims to add to the current veterinary literature on two opportunistic bacterial species (*Enterococcus hirae* and *Enterobacter xiangfangensis)* associated with bloodstream infections in small animals admitted to the Bologna University Veterinary Hospital.

**Case presentation:**

In the first case, a 15-year-old, immunocompromised, cardiopathic dog was admitted to the hospital for anorexia and diarrhea. The patient had a history of previous surgery and hospitalization. After three days, hyperthermia, leukopenia and hyperlactatemia were recorded, and blood culture revealed positivity for *Enterococcus hirae*, identified using matrix-assisted laser desorption/ionization time-of-flight mass spectrometry (MALDI-TOF MS). The patient’s general conditions progressively worsened, and the patient was euthanized. In the second case, a 2-year-old cat with chronic ocular herpesvirus infection and hypertrophic cardiomyopathy was admitted to the hospital for anorexia and hyperthermia. The cat was hospitalized one week before and received antimicrobial treatment for urinary tract infection by *Staphylococcus felis*. Hypokalemia and lymphopenia were also diagnosed. The patient progressively improved and was discharged after three days. On the same day, blood culture taken at admission revealed positivity for *Enterobacter xiangfangensis*, identified using MALDI-TOF MS. After five days, the patient returned with neurological symptoms, hypothermia and bradycardia, and was euthanized.

**Conclusions:**

In small animal veterinary practice, the impact of opportunistic bacterial agents (such as *E.hirae* and *E.xiangfangensis*) on bloodstream infections remains unclear. As in human medicine, they can be contracted in every healthcare setting and considered hospital-acquired infections. In this report, we highlighted the threat they pose especially in patients with multiple risk factors. Rapid and accurate diagnostic tools (such as MALDI-TOF MS) could be particularly important for reducing the severity of the infections.

## Background

Bloodstream bacterial infections are a common finding in both human and veterinary medicine and are considered an important cause of morbidity and mortality [1, 2]. Bloodstream infections not secondary to an infection at another body site are suggestive of a hospital-acquired infection (HAI), normally associated with the presence of intravascular devices such as central venous catheters [3]. Catheter-related bloodstream infections (CRBIs) are a frequent complication in immune-compromised and hospitalized patients with a high mortality rate. Microorganisms responsible for CRBI, other than well-known species such as *Escherichia coli, Klebsiella pneumoniae* or *Staphylococcus* spp., might include uncommon bacterial species, mostly commensal or opportunistic [4]. In small animal veterinary medicine, few epidemiological data are available concerning the role of environmental or commensal bacteria in the development of bloodstream infections in hospitalized patients. The aim of this report is to describe two clinical cases of bloodstream infections associated with opportunistic bacterial species in small animals hospitalized at the Bologna University Veterinary Hospital (UVH) of the Department of Veterinary Medical Sciences.

## Cases description

First case. A mixed breed of 15 years old female spayed dog weighing 16 kg was presented at the emergency service of the Bologna UVH for acute onset anorexia and diarrhea. On physical examination the dog was depressed with nausea, mild dehydration and a normal rectal temperature (38.5 °C). Blood profile showed lymphopenia (580 cells/mm^3^), mild hyperbilirubinemia (0.47 mg/dL), and a serum C-reactive protein (CRP) concentration within normal limits (0.9 mg/dL). Ultrasound examination was suggestive of an acute gastroenteritis with delayed gastric emptying. The dog had a past diagnosis of a subcutaneous mast cell tumor to the cranial tibial plate with involvement of the regional lymph nodes, which was surgically removed two months before, and was put on treatment with 2,5 mg/m^2^ vinblastine every three weeks (Velbe, EG Stada) and 1 mg/kg q 24 h prednisolone (Prednicortone, Dechra). Furthermore, the patient was affected by a myxomatous degeneration of both mitral and tricuspid valves and was on cardiologic therapy with 0.25–0.5 mg/kg q 24 h benazepril (Fortekor, Elanco), 0.25–0.5 mg/kg q 12 h pimobendan (Vetmedin, Boehringer Ingelheim) and 2 mg/kg q 12 h furosemide (Diuren, Teknofarma). Gastrointestinal (GI) toxicity by vinblastine was suspected based on history and clinical presentation and the dog was hospitalized in the general ward for monitoring and supportive treatment. On the third day of hospitalization, the dog developed a severe febrile neutropenia (rectal body temperature: 40,3 °C; white blood cell (WBC) count: 750 cells/mm^3^; neutrophil count: 230 cells/mm^3^) associated with ongoing GI signs. Blood chemistry revealed a significant increase in serum CRP (25.72 mg/dL) and onset of azotemia (creatinine: 1.77 mg/dL), indicative of acute kidney injury. Blood culture was then performed by inoculating 10 ml of blood into a commercially available blood culture bottle (Signal Blood Culture System; Oxoid, Milan, Italy) incubated at 37 ± 1 °C. The patient was then transferred to the Intensive Care Unit and empirical broad-spectrum antimicrobial treatment with piperacillin-tazobactam (50 mg/kg, IV q 6 h) was started. At 24 h, the blood culture was positive for the presence of microorganisms in the bloodstream. After subculturing at 37 ± 1 °C for 24 h in media plates for aerobic and anaerobic bacteria, *Enterococcus hirae* was identified. Bacterial identification was performed using the matrix-assisted laser desorption-ionization time-of-flight mass spectrometry method (MALDI-TOF MS;Biotyper, Bruker Daltonics, Billerica, MA), following manifacturer’s instructions (Bruker Daltonik, Bremen, Germany), with a final ID score of 2.35 (high-confidence identification) using the BRUKER BIOTYPER version 3.0 software. General clinical condition progressively worsened with persistent hyperthermia (40 °C) and onset of septic shock requiring hemodynamic support with vasopressors. In the following hours, multiorgan failure developed, and owners elected to euthanized their dog. Antimicrobial susceptibility testing of the isolated strain was performed using the Kirby–Bauer disk diffusion method, according to Clinical and Laboratory Standards Institute guidelines [5] and revealed resistance against clindamycin and rifampicin over a total of 17 antimicrobials tested.

Second case. A domestic, short-hair of 2 years old male castrated cat, weighing 5 kg, was referred to the Bologna VUH by the attending vet for the onset in the last week of lethargy, anorexia, vomiting and behavioral changes (hiding). The cat was initially treated and hospitalized in the referring practice, where a diagnosis of urinary tract infection by *Staphylococcus felis* was made and treatment with 2 mg/kg q 24 h marbofloxacin (Marbocyl, Vetoquinol) was instituted. Past history included chronic ocular herpesvirus infection treated with antiviral medication and a diagnosis of hypertrophic cardiomyopathy. At the hospital, the cat presented with dull mentation and hyperthermia (39.6 °C). blood culture was performed at admission by inoculating 5 ml of blood in the same commercially available bottle (Signal Blood Culture System; Oxoid, Milan, Italy) incubated at 37 ± 1° C. Blood examination results were unremarkable, except for an increased Serum Amyloid-A concentration (153 µg/mL) and positive test for Feline Leukemia Virus. During hospitalization, supportive treatment was instituted and intravenous marbofloxacin was continued. Clinical condition improved and the patient was discharged after three days. However, blood culture revealed positivity for *Enterobacter xiangfangensis*, identified after subculture at 37 ± 1° C for 24 h in media plates for aerobic and anaerobic bacteria, using MALDI-TOF MS with a final ID score of 2.41 (high-confidence identification). The isolated strain was subjected to antimicrobial susceptibility testing performed using the Kirby–Bauer disk diffusion method according to Clinical and Laboratory Standards Institute guidelines [5], and it showed resistance against all 14 tested antimicrobials, with the exception of amikacin and gentamycin. After 5 days, the patient was presented to the neurology service with severe neurological signs (stupor, pleurothotonus, head tilt, non-ambulatory tetraparesis, absent menace response, anisocoria), hypothermia (37.6 °C) and bradycardia (120 beats/min). A multifocal disease of inflammatory/infectious origin affecting the forebrain and brainstem was primarily suspected. A Magnetic Resonance Imaging was initially considered as a further diagnostic step, but due to the rapid deterioration of clinical signs, the cat was euthanized with the owner’s consent.

## Discussion and conclusions

In this study, we describe two cases of bloodstream infections associated with opportunistic bacterial agents in immunocompromised companion animals. Studies suggest that enteric or environmental bacteria can access the bloodstream through contamination of intravascular devices such as jugular and intravenous catheters [6, 7]. Such contamination can be associated with poor skin preparation or contaminated personnel’s hands or materials used in skin preparation [8, 9]. The catheter site should always be considered a potential source of infection if hyperthermia is registered and may occur without any externally detectable alteration of the site, mostly because of the intraluminal colonization of bacteria [10, 11]. Indeed, a study from human medicine by Safdar and Maki suggests that local inflammation is a rare event, and site appearance may not be predictive for the presence of CRBI [12]. In the presented cases, no inflammation was observed at the insertion site of the catheter. Opportunistic bacteria causing bloodstream infections can also have a urinary source or arise from the gastroenteric tract in patients with intra-abdominal pathologies or neutropenia. None of the cases described in this study presented additional positive bacteriological cultures from other body districts, and no further investigation of the source of the infection was performed. A positive blood culture for a bacterial species that is part of the normal cutaneous flora should be not considered as significant pathogen in an asymptomatic patient because of the potential contamination from the skin during blood sampling, but on the other hand any bacterial species that have virulence factors can potentially cause bacteremia or sepsis, especially in immunosuppressed patients [13, 14]. Although routine culture of catheters is not recommended, the present study has the limitation that a deeper investigation of the source of the septic picture, such as a bacteriologic culture of the catheter tip or insertion site, was not conducted. Also, autopsies and histopathological examinations were not performed in both cases, so a definitive diagnosis was not possible. Nevertheless, the association of a positive blood culture with clinical symptoms such as hyperthermia, tachycardia or bradycardia in patients with multiple risk factors suggests that it should be considered a real ongoing infection rather than contamination. In addition, both cases can be considered potential HAIs, following the definition given by Haque et al. [3]. To our knowledge, this report is the first to describe *E. hirae* and *E. xiangfangensis* as bloodstream infection agents in dogs and cats, respectively. In the first case, bacteremia caused by *Enterococcus hirae* was described in a dog. *E.hirae* is a gram-positive bacterium, commonly considered part of the fecal microbiota in animals such as bovines [15], poultry [16], dogs and cats [17, 18]. According to published reports, in humans it is rarely associated with infections, but the few cases always reported severe illnesses, such as acute pyelonephritis [19], endocarditis [20] spontaneous bacterial peritonitis [21] and acute cholecystitis [19], always associated with bacteremia, and in some cases with septic shock [22]. Moreover, Bourafa et al. [23] reported a case report of a urinary tract infection in a man caused by *E.hirae*, suggesting its potential role as ascending bacteria. The potential zoonotic transmission of the agent from cats to humans has been suggested by Blaseg and Hoover [24], reporting a wound infection caused by *E. hirae* associated with cat litter. In livestock animals, outbreaks in broiler flocks have been associated with *E. hirae*, which was isolated from sucking rabbits with diarrhea [25]. In companion animals, it has been described by van Loon et al. [26] as a bloodstream infection agent in a cat with bacteremia and endocarditis, but also as a cause of cholangitis and pancreatitis [27] and as an opportunistic intestinal pathogen in kittens [28], although Ghosh et al. [29] demonstrated that it is more commonly isolated in healthy kittens rather than in sick ones.

In the presented case, given the bacteremia associated with *E. hirae*, it can be considered as an opportunistic infectious agent in a patient with multiple risk factors (age, immunodeficiency, previous hospitalization). In the second case, bacteremia in a cat was sustained by *E. xiangfangensis*, a gram-negative bacterial species part of the *Enterobacter cloacae* complex (ECC). These bacteria are saprophytic in the environment and are also part of the gut microbiota of humans and animals [30–32], but they are also often related with HAIs in humans [33]. It was firstly described in 2014 as a new taxonomic species [34] and is associated with the production of antimicrobial-resistance enzymes such as metallo-β-lactamase, methylases [35] and carbapenemases [36]. According to Wu et al. [37], *E. xiangfangensis* was the dominant *Enterobacter* species isolated from 48 bloodstream infections at Sichuan University Hospital, and was associated with higher death rate, longer hospitalizations, and higher resistance to different antimicrobial classes. Its potential relation with neonatal sepsis has also been suggested [38]. Its relatively recent classification has led to a lack of case descriptions in veterinary medicine. It has been isolated from the intestinal tract of healthy armored catfishes (*Pterygoplichthys multiradiatus*) [39], and spontaneously fermented dairy products [40]. In companion animals, Daniels et al. [41] described *E. xianfangensis* as urinary tract and wound infection agent in two dogs, in both cases carrying carbapenemase genes. In the present case, bacteremia associated with *E. xiangfangensis* appeared in a patient with risk factors like immunodeficiency and previous hospitalization. Bacterial identification was possible in both cases due to the MALDI-TOF MS technology, which is an extremely effective tool in terms of rapidity and accuracy in laboratory diagnostics. Indeed, this technology is faster and more accurate than conventional phenotypic identification methods [42], leading to a species-level identification within minutes starting from an isolated bacterial colony. Compared with genotypic diagnostic methods, such as PCR or 16 S rRNA sequencing, it is more rapid, giving the chance to reduce severe clinical outcomes, and requires less technical demands, normally available only in reference laboratories. In conclusion, this study highlights the role of *E.hirae* and *E.xiangfangensis*, two opportunistic bacterial species, as agents of bacteremia in companion animals. Bloodstream infections caused by these agents can often be contracted in any healthcare setting, especially in patients with risk factors, leading to sepsis and poor outcomes, such as in the described cases. Their impact as opportunistic agents and their infection rates in small animal veterinary medicine have probably been underestimated in the past years and should be further investigated. Considering the threat they pose, a rapid and accurate diagnostic method (such as MALDI-TOF MS) for their identification might be particularly important to reduce the possibility of poor prognosis.

## Data Availability

Data sharing is not applicable to this article as no datasets were generated or analyzed during the current study.

## List of abbreviations

MALDI-TOF MS: matrix-assisted laser desorption/ionization time-of-flight mass spectrometry
HAI: Hospital-Acquired Infection
CRBI: Catheter-Related Bloodstream Infection
UVH: University Veterinary Hospital
CRP: serum C-reactive protein
GI: gastro-intestinal
WBC: white blood cells
ECC: *Enterobacter cloacae* complex
